# The effects of anatomical errors on shoulder kinematics computed using multi-body models

**DOI:** 10.1007/s10237-022-01606-0

**Published:** 2022-07-22

**Authors:** Maxence Lavaill, Saulo Martelli, Luke Gilliland, Ashish Gupta, Graham Kerr, Peter Pivonka

**Affiliations:** 1 School of Mechanical, Medical and Process Engineering, Queensland University of Technology, Brisbane, QLD Australia; 2 Queensland Unit for Advanced Shoulder Research, Brisbane, QLD Australia; 3 Medical Device Research Institute, College of Science and Engineering, Flinders University, Tonsley, SA Australia; 4 Movement Neuroscience Group, School of Exercise & Nutrition Sciences, Queensland University of Technology, Brisbane, QLD Australia; 5 Greenslopes Private Hospital, Brisbane, QLD Australia

**Keywords:** Shoulder kinematics, Anatomical errors, Multi-body model, Scaling, Image-based

## Abstract

Joint motion calculated using multi-body models and inverse kinematics presents many advantages over direct marker-based calculations. However, the sensitivity of the computed kinematics is known to be partly caused by the model and could also be influenced by the participants’ anthropometry and sex. This study aimed to compare kinematics computed from an anatomical shoulder model based on medical images against a scaled-generic model and quantify the effects of anatomical errors and participants’ anthropometry on the calculated joint angles. Twelve participants have had planar shoulder movements experimentally captured in a motion lab, and their shoulder anatomy imaged using an MRI scanner. A shoulder multi-body dynamics model was developed for each participant, using both an image-based approach and a scaled-generic approach. Inverse kinematics have been performed using the two different modelling procedures and the three different experimental motions. Results have been compared using Bland–Altman analysis of agreement and further analysed using multi-linear regressions. Kinematics computed via an anatomical and a scaled-generic shoulder models differed in average from 3.2 to 5.4 degrees depending on the task. The MRI-based model presented smaller limits of agreement to direct kinematics than the scaled-generic model. Finally, the regression model predictors, including anatomical errors, sex, and BMI of the participant, explained from 41 to 80% of the kinematic variability between model types with respect to the task. This study highlighted the consequences of modelling precision, quantified the effects of anatomical errors on the shoulder kinematics, and showed that participants' anthropometry and sex could indirectly affect kinematic outcomes.

## Introduction

Measurement of shoulder kinematics is essential for evaluating upper limb functional impairment resulting from injury (Wang et al. 2001), movement disorders (Kibler et al. 2012), and for determining the effectiveness of movement coordination in sports (Kibler 1998) and in the workplace (Linaker et al. 2015). It is also critical for assessing functional outcomes of shoulder surgery and rehabilitation (McMullen et al. 2000). However, experimental errors and differences in computational methods can affect the calculation of shoulder kinematics, limiting the validity of information available in the clinical decision-making process.

Shoulder kinematics is most often calculated from skin-mounted marker’s trajectories recorded using motion capture, i.e. the method is called direct kinematics (DirectK) (Lempereur et al. 2014; Charbonnier et al. 2014). However, the direct use of skin-marker trajectories is known to introduce errors regarding the exact identification of bony landmarks (De Groot 1997) and artifacts arising from skin motion (Begon et al. 2015), particularly for tracking the scapula motion due to large layers of soft tissues overlying the bone. The use of a kinematic scapular measurement device limits errors to less than 3 degrees on average for every scapular angle; however, the latter errors can double or triple above 90 degrees of shoulder elevation (Warner et al. 2012). Once marker trajectories are captured, joint angles can be directly inferred by evaluating a segment’s Euler/Cardan orientation with respect to a parent (Senk et al. 2006). The results are known to be highly dependent on the sequence used and are sensitive to marker trajectory errors. Finally, the direct method does not try to satisfy joint congruency, which can result in unrealistic joint distractions or co-penetrations (Bourgain et al. 2018).

Another way to compute shoulder kinematics is using inverse kinematics (IK) computation and a multi-body dynamics shoulder model that imposes joint congruency (Bolsterlee et al. 2013, Cheze et al. 2015, D’Souza et al. 2001, Duprey et al. 2017). Using this method, the orientation of each body/segment is determined by minimising the global marker errors at each frame of a movement (Delp et al. 2007, Cheze et al. 2015). A comprehensive comparison between DirectK and IK was achieved in the lower limbs and showed small differences (Kainz et al. 2016). Overall, the global optimisation is known to limit the impact of skin motion artifacts and marker misplacement (Lu and O’Connor 1999). However, the IK process strongly depends on the accuracy of anatomical points, such as the model’s joint locations, and on the imposed motion constraints.

Few generic upper-limb models are available and widely used in the scientific community to perform IK (Nikooyan et al. 2010; Wu et al. 2016; Seth et al. 2016; Damsgaard et al. 2006, Charlton and Johnson 2006). Personalisation of these models is commonly undertaken by linearly scaling the generic bony shapes to match targeted anatomical measurements from a subject (Nikooyan et al. 2010, 2011; Wu et al. 2016). However, linear scaling factors may not be sufficient to describe the complexity of the shoulder bony structures (e.g. scapula) and may lead to anatomical errors. Then, in turn, these can translate into inaccuracies in the model bony landmarks and coordinate systems and propagate to the computation of joint angles, as shown in lower limb studies (Koller et al. 2021).

Furthermore, Klemt et al. (2019) highlighted the possibility that some anthropometric features, not directly accounted in usual scaling processes (e.g. body mass index (BMI), mass, full height, ratio of body height to shoulder width) could indirectly be associated with variations in the model architecture and thus in the modelled kinematics. Anthropometry of the baseline model should therefore be accounted for before scaling, and this impact needs to be studied further.

In contrast to generically scaled models, models can also be personalised from direct measurements of medical images. So-called subject-specific image-based models are becoming increasingly popular (Martelli et al. 2011; Valente et al. 2017; Klemt et al. 2019; Charbonnier et al. 2014) and are driven by the hypothesis that more accurate capturing of bony anatomy leads to more accurate computational models. With this modelling modality, anatomical errors (i.e. errors in the joint-to-joint distances) and the definition of joint coordinate systems are directly influenced by the resolution of the image used. Such errors are usually in the millimetre range for typical medical imaging modalities (i.e. computed tomography and magnetic resonance imaging) (Nowogrodzki 2018, Lin and Alessio 2009).

Several studies investigated the effect of errors committed while fitting a generic musculoskeletal model to a participant by focusing on a variety of musculoskeletal factors ranging from joint angles to muscle forces (Valente et al. 2014; Martelli et al. 2015a, b; Kainz et al. 2016; Martelli et al. 2020; Koller et al. 2021). Moreover, studies have compared scaled-generic and image-based models (Martelli et al. 2015a, b; Kainz et al. 2021). However, all of these studies focused on the lower limb, while the effect of anatomical scaling errors on shoulder kinematics is yet poorly understood. The performance of personalised models has never been compared to those of generic models for the shoulder. Furthermore, no study has been undertaken on how the anatomical errors propagate to the computation of the shoulder joint kinematics. We hypothesised that different personalisation procedures of the same individual would produce significantly different kinematics based on variations in the kinematic chain but also influenced by the individual’s anthropometry.

The aim of this work was to develop and study two different modalities of shoulder models, i.e. an image-based, anatomically correct model and a scaled-generic (SG) model, and to report their kinematic outcomes obtained using experimental shoulder motions. Overall, we aimed to answer the following research questions:

- Do different modelling modalities significantly vary the computed shoulder kinematics?
- How much do the kinematics vary with anatomical errors?
- Other than the anatomical errors, does anthropometry play a role in the kinematic outcomes?

## Methods

### Participants

Twelve healthy volunteers (8 females, 4 males, age: 28.6 ± 4.3 yrs, weight: 60.0 ± 11.4 kg, height: 167.0 ± 7.9 cm) with no history of shoulder pain or pathology were recruited for this study. Ethics approval was obtained from the QUT Human Research ethics committee, and all participants provided written informed consent prior to participation (QUT ethics #2,000,000,470). Participants’ information about their age, sex, height, and weight were collected. Each participant underwent an MRI scan of the upper arm and shoulder and participated in a motion capture recording of upper limb movement on separate days.

### Imaging

Hemithorax (including cervical/thoracic spine and sternum) and dominant upper extremity were imaged using a 3 Tesla MR scanner (Ingenia, Koninklijke Philips N.V., The Netherlands) at voxel sizes of 0.4 × 0.4 × 0.8 mm (using a T1 Dixon sequence). The sternum, clavicle, scapula, and humerus in the images were then segmented using Mimics 23.0 (Materialise, Leuven, Belgium) to create a 3D anatomical model.

### Experimental motions

Seven retro-reflective markers were attached to relevant anatomical landmarks of the thorax (C7, T8, IJ, PX), right shoulder (AC), and arm (EL, EM) according to the ISB standards (cf. Wu et al. 2005 for anatomical landmark’s abbreviations). A marker cluster was placed on the flat bony part of the scapular spine and used to track scapula motions as well as average and limit skin motion artifacts (Warner et al. 2015; Matsui et al. 2006). Other scapular landmarks (AA, TS, AI, PC) were then located in the scapular coordinate system using a registration wand (Warner et al. 2015). Lastly, a marker cluster was attached to the participant’s dominant upper arm, and participants were asked to perform shoulder circumduction for 30 s. The latter task allowed us to determine the location of the functional glenohumeral joint (GHJ) centre in the humeral head using the SCoRE method (Ehrig et al. 2006).

The participants were instructed to stand and perform six repeated trials of three different shoulder planar motions, i.e. shoulder abduction/adduction (AA), flexion/extension (FE) and internal/external rotation (IER). AA and FE tasks were executed up to shoulder level (approximately 90° of elevation) to limit skin-motion artifacts at the scapula cluster (Warner et al. 2015). The tasks were performed starting with the arm along the thorax and the elbow extended and were undertaken in three consecutive phases:

- Phase 1: arm elevation to reach shoulder level (approximately 90° between thorax and arm),
- Phase 2: arm staying still at shoulder level,
- Phase 3: arm depression to reach initial posture.
  The IER task was performed starting with the elbow flexed at 90 degrees and the shoulder at maximal external rotation and was undertaken in three consecutive phases:
- Phase 1: arm internal rotation to reach contact with the thorax,
- Phase 2: arm staying still at thorax contact,
- Phase 3: arm maximal external rotation to reach initial posture.

All trials were performed using a metronome, each phase performed over a time period of 2 s. Marker trajectories were recorded at 200 Hz using a 12-camera motion capture system (Vicon Motion Systems, Oxford, UK) and filtered using a second-order, zero-lag, 4 Hz low-pass Butterworth filter (Nikooyan et al. 2010).

GHJ angles were computed in *MATLAB* directly from experimental markers using joint coordinate systems based on the ISB (Wu et al. 2005). Moreover, in order to avoid frequent gimbal lock issues, we computed the kinematics using a YXZ Cardan sequence at the GHJ, as recommended by Šenk and Chèze (2006)In the following, this direct kinematics is denoted as DirectK.

### Multi-body modelling

The model selected for the study was structured as a four segment, eight degrees-of-freedom (DOF) multi-body model of the upper limb. The sternoclavicular joint (SCJ) was modelled as a two DOF universal joint, and both the acromioclavicular joint (ACJ) and GHJ as three DOF ball-and-socket joints.

**Fig. 1:**
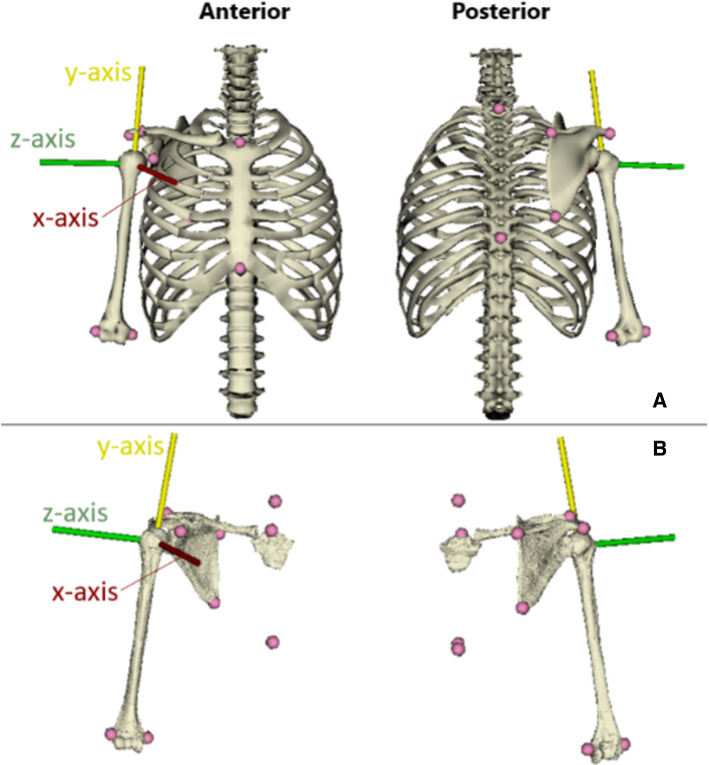
SG model (**a**) and MRI-based model (**b**) of the right shoulder – Also shown is the parent coordinate system of the GHJ. Each motion is calculated from the Cardan sequence YXZ

On the one hand, an anatomical model was designed from segmented MR images of the sternum, clavicle, scapula, and humerus bones to create an image-based subject-specific model of each participant. In the following, the latter model is denoted as MRI-based model (cf. Figure 1).

On the other hand, a more common modelling method was adopted and used generic bone shapes, available in the *OpenSim* package (Delp et al. 2007), to create a generic shoulder model.

For both modelling procedures, each bony landmark was digitally selected on the virtual bone surfaces by a trained operator using the open-source software *NMSbuilder* (cf. Figure 1) (Martelli et al. 2011; Valente et al. 2017). The model-based GHJ centre was determined as the centre of the best-fit sphere onto the humeral head (Meskers et al. 1998). Bony landmarks and joint coordinate systems were defined based on the ISB recommendations and the previously described Cardan sequence YXZ. (Wu et al. 2005, Šenk and Chèze 2006).

Furthermore, the generic model was subsequently scaled to match the anthropometric measurements of each participant. The clavicle and humerus segments were linearly scaled using joint-to-joint distances, while two measurements were used to scale the scapula (between AC and TS and between AI and TS) due to its complex shape. Finally, the thorax segment was scaled in three different directions. In the following, this model is denoted as SG model (cf. Figure 1).

Anatomical errors were calculated as the difference in inter-joint distances, i.e. distances between SCJ and ACJ, between ACJ and GHJ and between GHJ and the elbow joint, between the MRI-based and the SG models. These anatomical errors are, respectively, noted as ${\Delta }_{\Vert Clavicle\Vert }$, ${\Delta }_{\Vert Scapula\Vert }$ and ${\Delta }_{\Vert Humerus\Vert }$ in the following.

Each model was utilised to perform IK using *OpenSim 3.3 API* in *MATLAB* with the six experimental trials of each motion. All marker weights were assigned as 1. Eventually, a global root-mean-square deviation (RMSd) tolerance of 2.5 cm was achieved between experimental and virtual (i.e. model) markers for all IK computations, as suggested by Delp et al. (2007). RMSd are reported in Supplementary material A. Joint angles from the whole shoulder complex (SCJ, ACJ, and GHJ) were computed and can be found in Supplementary material B. However, for the readability of this paper, it was decided that only the angles from the GHJ would be reported in the core of the paper, as a representation of the whole kinematic chain.

### Data analysis

For each shoulder motion, time was linearly normalised using a custom-made *MATLAB* script, with each motion phase (described in Sect. 2.3) corresponding to one-third of the normalised task.

The main shoulder angle in each of the three tasks was analysed (i.e. around *X*-axis, around *Z*-axis, around *Y*-axis of the scapula coordinate system, for the AA, FE, IER tasks, respectively). The mean and standard deviation of the GHJ angles were computed based on the 6 repeated trials of each shoulder motion. Kinematics computed using the SG and MRI-based models were also compared against DirectK.

The difference in the kinematics of the two modelling procedures due to anatomical errors was analysed using a Bland–Altman analysis of agreement. This analysis displays the relationship between the difference and the average of two measurement methods. It assumes that the differences between the two methods follow a normal distribution. Based on this assumption, 95% of the differences lie between the limits of agreement (LOA), representing two fold its standard deviation (Bland and Altman 1986).

Kinematics between the DirectK and the SG model as well as the DirectK and the MRI-based model were compared using Bland–Altman plots. The method suggested by Bland and Altman was applied (Bland and Altman 1995, 2003) to compute the 95% LOA. Moreover, the mean difference, known as bias, was plotted and represented the systematic difference between the two methods. The bias indicates whether, on average, one method tends to underestimate or overestimate measurements relative to the other method.

Furthermore, a multiple linear regression model was developed to quantify the influence of participants’ anthropometric measurements and anatomical errors on kinematic differences, represented by the difference of maximum range of motion (ΔROM). The predictors of the regression model were selected to be the modelling anatomical errors (cf Sect. 2.4) and the participant’s sex and BMI, as they either presented significant correlation during preliminary simple linear regressions or due to their clinical significance (see Eq. (1)).

1 $$\Delta \mathrm{ROM }=\mathrm{Intercept }+ {\upbeta }_{1}\times \mathrm{Sex }+ {\upbeta }_{2}\times \mathrm{BMI }+{\upbeta }_{3}\times {\Delta }_{\Vert \mathrm{Clavicle}\Vert } + {\upbeta }_{4}\times {\Delta }_{\Vert \mathrm{Scapula}\Vert } + {\upbeta }_{5}\times {\Delta }_{\Vert \mathrm{Humerus}\Vert }$$

Sex was considered a categorical predictor where males and females correspond to the values 1 and 1.1, respectively. An accurate regression model was assumed to present a p-value of 0.05 or lower. Moreover, a predictor’s impact on the regression model was considered significant with a t-stat of 2 or higher.

## Results

**Fig. 2:**
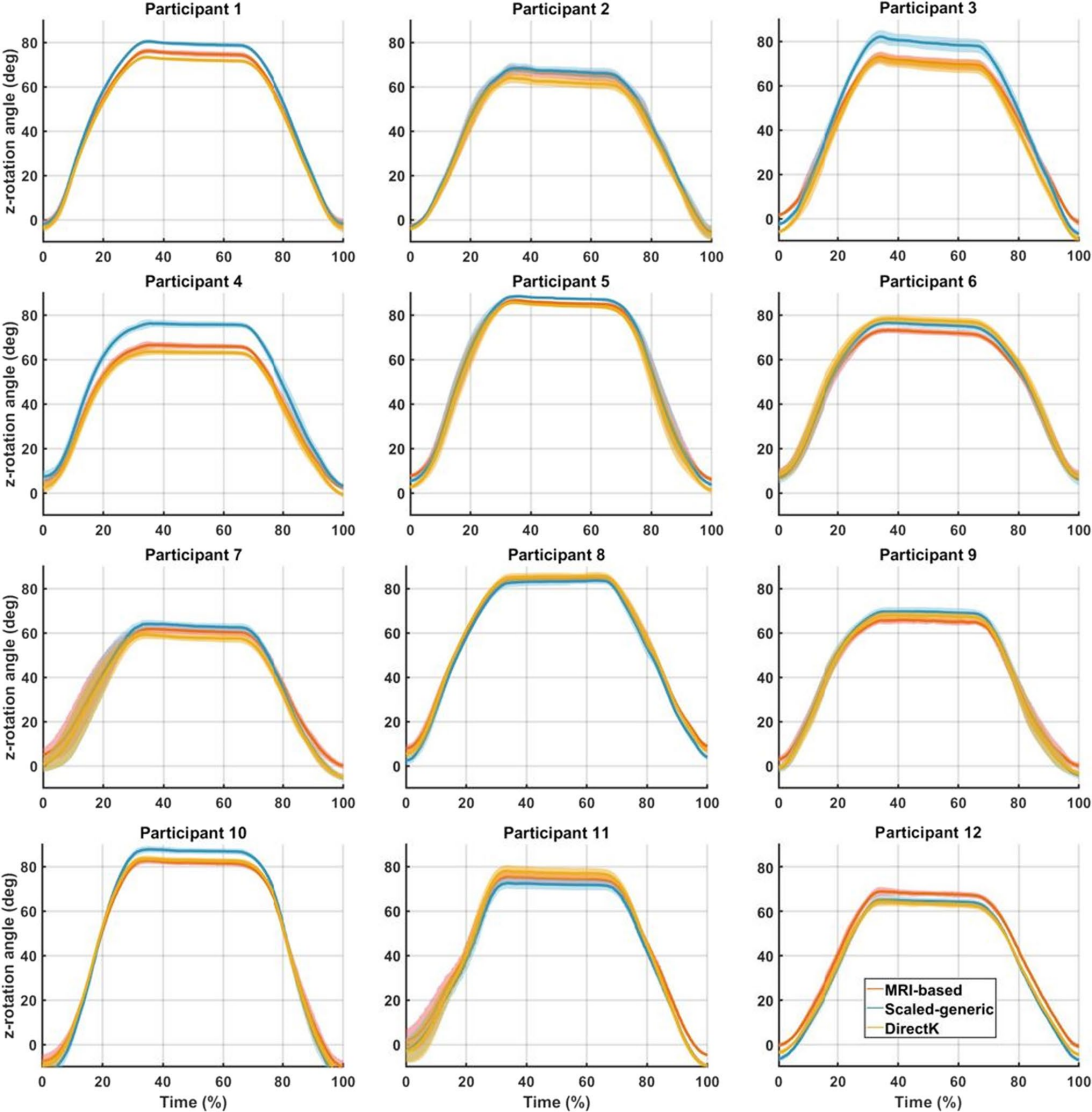
Shoulder Z-angle against normalised FE task. Results from each participant’s MRI-based model, SG model and DirectK are plotted in orange, blue, and yellow, respectively. Solid line and area represent respectively the mean and standard deviation of the six repeated trials

Kinematic outcomes of the 12 participants during the FE task are shown in Fig. 2. Means and standard deviations are calculated and plotted for the MRI-based model, the SG model and DirectK. Standard deviations are representative of the intra-subject variability of the task. Every participant presented differences in kinematics between the MRI-based and the SG models. Participant 4 demonstrated the greatest disparity between models (RMSd =  ± 8.0°). On average, the RMSd between models was ± 3.8° for the FE task. Similar results are included in Appendices A and B for the AA and the IER tasks, respectively. On average, the RMSd between models was ± 5.4° and ± 3.2° for the AA and IER tasks, respectively.

**Fig. 3:**
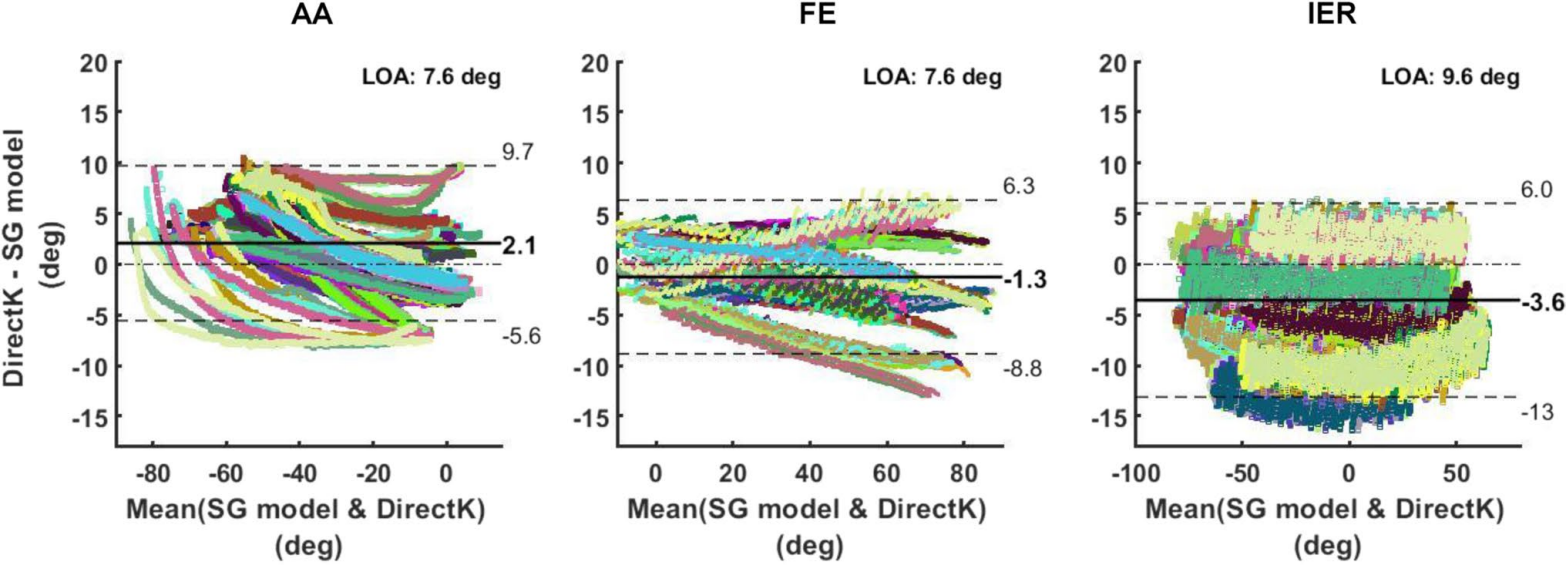
Bland–Altman analysis of agreement reporting the agreement between DirectK and the SG model in terms of the main rotation angle of the different shoulder tasks, i.e. AA task (left column), FE task (middle column) and IER task (right column). A solid black line represents the bias between the techniques. Dotted lines represent the mean ± the limits of agreement. Each colour represents one repetition for one participant

Figure 3 shows the Bland–Altman analyses of agreement between the kinematics computed via DirectK and the SG model. Minimum and maximum LOAs were −5.6° to 9.7° for AA, −8.8° to 6.3° for FE, −13.0° to 6.0° for IER tasks, respectively. Biases were 2.1° for AA, −1.3° for FE, and −3.6° for IER between the DirectK and SG model joint angles.

**Fig. 4:**
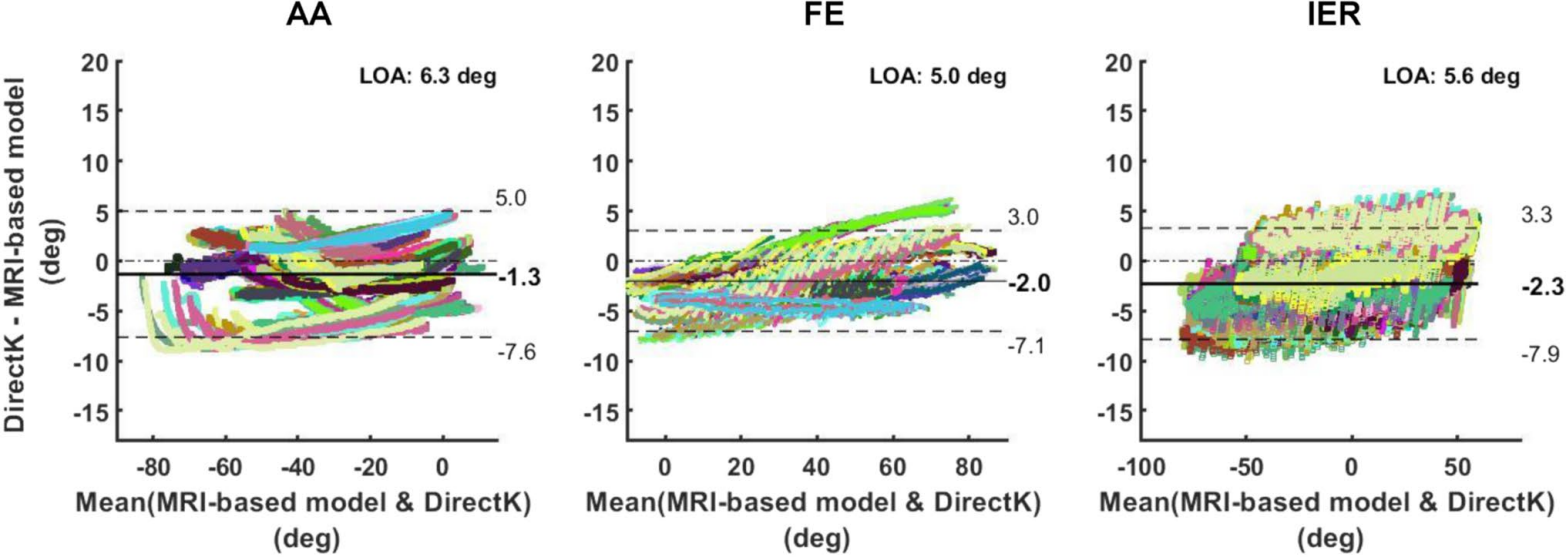
Bland–Altman analysis of agreement reporting the agreement between DirectK and the MRI-based model in terms of the main rotation angle of the different shoulder tasks, i.e. AA task (left column), FE task (middle column) and IER task (right column). A solid black line represents the bias between the techniques. Dotted lines represent the mean ± the limits of agreement. Each colour represents one repetition for one participant

Figure 4 shows the Bland–Altman analyses of agreement between the kinematics computed via DirectK and the MRI-based model. Minimum and maximum LOAs were −7.6° to 5.0° for AA, −7.1° to 3.0° for FE, and −7.9° to 3.3° for IER tasks. Biases were −1.3° for AA, −2.0° for FE and −2.3° for IER between joint angles from DirectK and MRI-based model.

Based on the multiple linear regression models, the differences in ROM computed via the MRI-based and SG models, and noted as $\Delta ROM$, is explained by anthropometric measurements and anatomical errors. Regression coefficients for each task are summarised in Table 1. Statistics are found in Table 2.

**Table 1:** Multi-linear regression models explaining $\Delta ROM$ between the MRI-based and SG models during each motion type – Regression coefficients

$\Delta ROM$	Intercept [°]	${\upbeta }_{1}$[°/(M/F)]	${\upbeta }_{2}$[°/(kg/m^2^)]	${\upbeta }_{3}$[°/cm]	${\upbeta }_{4}$[°/cm]	${\upbeta }_{5}$[°/cm]	R^2^	Adjusted R^2^	*p*-value
AA	− 5.6	1.3	0.9	1.5	5.0	− 8.9	0.89	0.80	0.008
FE	− 8.2	− 2.3	− 0.7	0.8	6.5	− 1.6	0.82	0.68	0.03
IER	− 28.6	− 5.8	1.7	0.6	2.6	− 1.8	0.68	0.41	0.14

ROMs were computed around the scapular *X*-axis, *Z*-axis, and *Y*-axis, for the AA, FE, and IER tasks respectively. ${\Delta }_{\Vert \mathrm{Clavicle}\Vert }$, ${\Delta }_{\Vert \mathrm{Scapula}\Vert }$ and ${\Delta }_{\Vert {\mathrm{Humerus}}\Vert }$ each represents the joint-to-joint distance differences between the MRI-based and SG models. Male corresponded to the categorical value 1, female corresponded to the categorical value 1.1. The multi-linear regression models were described as $\Delta ROM =\mathrm{Intercept} + {\beta }_{1}\times Sex + {\beta }_{2}\times BMI +{\beta }_{3}\times {\Delta }_{\Vert \mathrm{Clavicle}\Vert } + {\beta }_{4}\times {\Delta }_{\Vert \mathrm{Scapula}\Vert } + {\beta }_{5}\times {\Delta }_{\Vert \mathrm{Humerus}\Vert }$

**Table 2:** Standard error (SE), t-statistic and precision on the multi-linear regression coefficients explaining $\Delta ROM$ between the MRI-based and SG models during each motion type

		Intercept [°]	${\beta }_{1}$[°/(M/F)]	${\beta }_{2}$[°/(kg/m^2^)]	${\beta }_{3}$[°/cm]	${\beta }_{4}$[°/cm]	${\beta }_{5}$[°/cm]	R^2^	Adjusted R^2^	*p*-value
AA	SE	10.1	1.9	0.5	0.8	1.5	1.5	0.89	0.80	0.008
	t-stat	− 0.6	0.7	1.8	1.8	3.5	− 5.9			
FE	SE	9.5	1.8	0.5	0.8	1.4	1.4	0.82	0.68	0.03
	t-stat	− 0.9	− 1.3	1.5	1.0	4.7	− 1.1			
IER	SE	25.0	4.8	1.2	2.1	3.6	3.8	0.68	0.41	0.14
	t-stat	− 1.1	− 1.2	1.4	0.3	0.7	− 0.5			

The multi-linear regression models were described as $\Delta ROM=\mathrm{Intercept}+ {\beta }_{1}\times Sex + {\beta }_{2}\times BMI +{\beta }_{3}\times {\Delta }_{\Vert \mathrm{Clavicle}\Vert } + {\beta }_{4}\times {\Delta }_{\Vert \mathrm{Scapula}\Vert } + {\beta }_{5}\times {\Delta }_{\Vert \mathrm{Humerus}\Vert }$

From Tables 1 and 2, the model predictors ${\Delta }_{\Vert Clavicle\Vert }$, ${\Delta }_{\Vert Scapula\Vert }$ and ${\Delta }_{\Vert Humerus\Vert }$, as well as the sex and the BMI explain respectively 80%, 68%, and 41% of the variance in ROM between the MRI-based and the SG models.

## Discussion

In this study, we compared the kinematics directly measured from experimental data (i.e. DirectK) with two different modelling techniques, one based on generic bony shapes and linear scaling factors and the other, more anatomically correct, based on a subject-specific representation of the participant’s anatomy from medical images.

Using the same experimental data as inputs, the angles computed via IK were significantly different using the two shoulder modelling approaches, as shown by the reported RMSd and $\Delta ROM$. These kinematic differences were expected as the two modelling procedures were different by nature, leading to systematic differences in the outputs. Noticeably, variations of up to 12% of the whole ROM could be observed depending on the technique (cf. Figure 2), although both model types represented the same individual. In addition to these variations due to the model used, one can expect errors of 1–3° in the ROM due to the global optimisation kinematic method with respect to the actual motion of shoulder bones as shown by Charbonnier et al. (2014).

Using an MRI-based model gave smaller biases. The LOAs to DirectK were smaller when using an MRI-based model than when using a SG model for the AA and FE tasks. In other words, kinematics computed via the MRI-based model is a closer representation of the experimental kinematics, independently of the individuals or the tasks studied. One can note that direct comparison between MRI-based and SG models can also be achieved by subtracting both results. As shown in Klemt et al. (2019), linear scaling is not sufficient to guarantee the similarity between two models which are originally different in bony shape. This explains why the SG model doesn’t achieve the same results as the MRI-based one.

Although the image-based model is theoretically an exact representation of the bony anatomy of an individual, its exactness still depends on the resolution of the medical scans, usually less than 0.1 mm (Nowogrodzki 2018, Lin and Alessio 2009), on the quality of bone segmentation, with errors around 0.5 mm (Van den Broeck et al. 2014) and on the virtual palpation of bony landmarks, with inter-operator 3D errors usually above 1 mm (De Groot 1997).

In the current study, DirectK is used as a reference, due to its more accessible approach, and compared against the kinematics computed using a rigid-body model. Skin-mounted markers present several known issues when dealing with kinematics, such as ongoing errors on marker placements and skin motion artifacts (De Groot 1997; Lempereur et al. 2014). IK computation along with the use of an anatomically exact model, such as the MRI-based model presented here, potentially correct the intrinsic errors of experimental markers. Indeed, by constraining the markers to an accurate representation of the participant’s skeleton, the IK process will limit marker trajectories to realistic, possible ranges, and therefore reduce kinematic errors.

The regression models developed in the current paper showed that differences in inter-joint distances explained most of the kinematic differences between models. These results confirm what has been reported in studies on lower limbs (Puchaud et al. 2020). Scapular and humeral lengths have the most significant influences on computed GHJ kinematics. Thus, these kinematic chain distances appear critical for defining any shoulder model and providing good outcomes of the IK process.

Moreover, participants’ sex and BMI likely play a role in the kinematic differences, although not currently significant (i.e. *t*-stat < 2). Their addition to the regression models increased the overall R^2^ and p-values compared to preliminary models. Sex could impact the precision of the SG model (Klemt et al. 2019) due to differences in skeletal anatomies (Merrill et al. 2009; Jacobson et al. 2015), and large BMI could be responsible for experimental skin motion artifacts (Gupta et al. 2013). However, a larger study would be necessary to address this point.

Kinematic errors have a significant impact on biomechanical studies. For instance, in rigid body dynamics, the outputs from inverse dynamics and static optimisation (Delp et al. 2007) are known to vary substantially when the kinematics is modified (Valente et al. 2014; Martelli et al. 2020; Kainz et al. 2021; Koller et al. 2021). Hence, it is critical to understand these errors and identify modifiable model parameters that can limit them.

Although considered as anatomically more accurate, personalised rigid-body models based on medical images are known to be more cost-, resource- and time-consuming to produce than generic models. Note, however, that this cost tends to decrease with progress made in the automation of medical imaging segmentation as well as the automatic setting of joint coordinate systems from virtually guided bony landmark palpation (this feature is programmed in *NMSbuilder* for the lower limbs, but not for the upper limbs) (Martelli et al. 2011; Valente et al. 2017).

It is worth noting that the study initially used the Euler sequence YX’Y’’ recommended by the ISB for calculating angles at the GHJ (Wu et al. 2005). Even though the latter sequence allows a clinical description of the GH motion (i.e. shoulder plane, elevation and rotation), its use caused gimbal lock issues when the arm was around 0°or 90° abduction. The Cardan sequence YXZ proposed by Šenk and Chèze (2006) allowed computation of GH kinematics with minor or no modification post-computation.

This study had some limitations. Concerning the selected motion tasks, this study analysed simple motions, i.e. lying within anatomical planes. The significance of the study could benefit from more complex, out-of-plane, shoulder motions reflective of everyday movements. Another limitation was that the subject cohort used in the present study did not introduce a large variance in height and weight. However, it still represented the Australian population mean (ABS 2018). No participant represented anthropometric extremes. Finally, the statistical significance of the Bland–Altman analysis and regression models could be improved with more participants.

## Conclusion

As per this study, we showed the significant difference in shoulder kinematics computed using two different modelling techniques, i.e. image-based and SG rigid-body models, and how the anatomical errors in the latter influenced the kinematics computation. Kinematics computed via an image-based model is a closer representation of DirectK than via SG models, and independently of the individuals or the tasks studied. Finally, the main reasons explaining the kinematic differences between modelling techniques were the differences in segment lengths. However, the sex and BMI of the studied participants could also influence the computation of joint angles.

## Figures and Tables

**Fig. 5:**
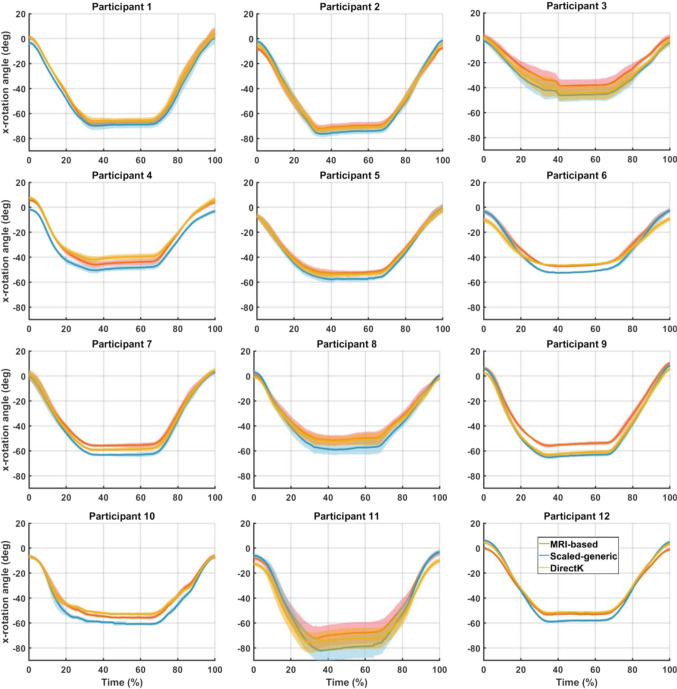
Shoulder X-angle against normalised AA task. Results from each participant's MRI-based model, SG model, and DirectK are plotted in orange, blue, and yellow, respectively. Solid line and area represent, respectively, the mean and standard deviation of the six repeated trials. Note that using the ISB standards, a shoulder abduction provides a negative X angle

**Fig. 6:**
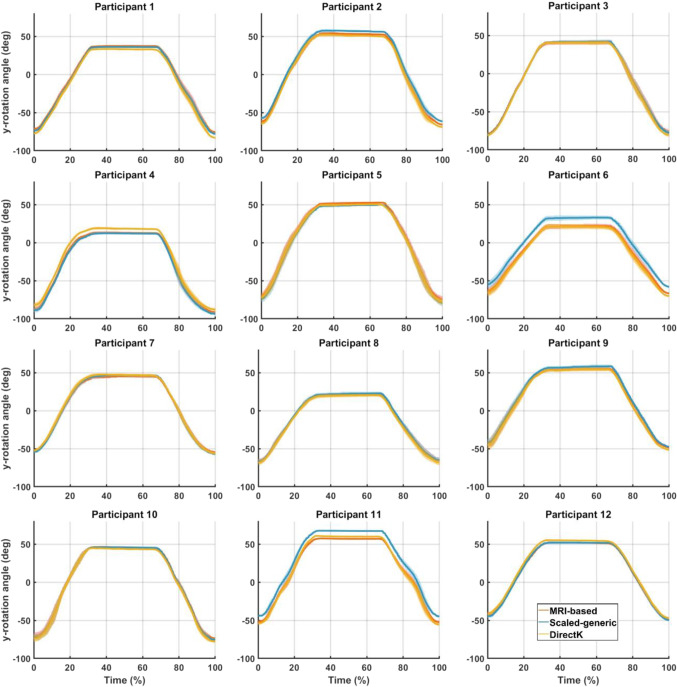
Shoulder Y-angle against normalised IER task. Results from each participant’s MRI-based model, SG model and DirectK are plotted in orange, blue, and yellow, respectively. Solid line and area represent, respectively, the mean and standard deviation of the six repeated trials

## Appendix

### Appendix A: Shoulder abduction – adduction task

See Fig. 5.

### Appendix B: Shoulder internal – external rotation task

See Fig. 6.
